# Styloid Process Elongation as an Incidental Finding in Adult Orthodontic Patients: Prevalence, Morphology and Diagnostic Implications from Panoramic Radiography

**DOI:** 10.3390/diagnostics16132097

**Published:** 2026-07-03

**Authors:** César Martínez-Rodríguez, Alfonso Alvarado-Lorenzo, José María Alamán-Fernández, Juan Santos-Marino, María Andrés-Veiga, Natalia Martínez-Rodríguez

**Affiliations:** 1 Department of Dental Clinical Specialties, Faculty of Dentistry, Complutense University of Madrid, 28040 Madrid, Spainnataliamartinez@ucm.es (N.M.-R.); 2 Department of Surgery, Faculty of Medicine, University of Salamanca, 37008 Salamanca, Spain; kuki@usal.es; 3 Faculty of Health Sciences, Alfonso X University (UAX), 28691 Madrid, Spain

**Keywords:** styloid process, elongation, panoramic radiography, orthodontics, skeletal class, facial pattern, incidental findings

## Abstract

**Background:** The styloid process exhibits considerable anatomical variability, and its elongation is frequently identified as an incidental finding on panoramic radiographs. However, limited evidence exists regarding its prevalence and possible association with craniofacial skeletal characteristics in adult orthodontic patients. Therefore, the aim of this study was to evaluate the prevalence, morphological characteristics, mineralisation patterns, and possible association of styloid process elongation with skeletal class and facial pattern in an adult orthodontic population. **Methods:** A retrospective cross-sectional study was conducted on 340 adult orthodontic patients (130 males and 210 females; mean age: 51.55 ± 6.62 years). Panoramic radiographs were used to assess styloid process elongation, defined as ≥30 mm, as well as its morphology and mineralisation patterns according to the Langlais classification. Lateral cephalograms were analysed to determine skeletal class and facial pattern. Statistical analysis included descriptive and inferential methods, with significance set at *p* < 0.05. **Results:** Styloid process elongation was identified in 47.65% of patients. Elongated processes showed significantly greater length and side asymmetry (*p* = 0.001). The most frequent morphology was normal (Type IV), while complete mineralisation (Type D) predominated. No significant associations were found between elongation and age or sex. Furthermore, no statistically significant relationship was observed between elongation and skeletal class (*p* = 0.479) or facial pattern (*p* = 0.531). Only a small proportion of patients reported symptoms according to the available clinical records, with no clear association with styloid process elongation. **Conclusions:** Styloid process elongation is a common incidental finding in adult orthodontic patients and does not appear to be associated with skeletal class or facial pattern. Its recognition on panoramic radiographs may improve the differential diagnosis of orofacial and cervical symptoms.

## 1. Introduction

The styloid process is a bony projection of the temporal bone whose length, morphology, and degree of mineralisation exhibit considerable anatomical variability. These variations have been studied for decades, leading to the development of different classifications based on morphological and radiographic characteristics. Among the earliest studies, Gossman and Tarsitano [1] described elongated, deviated, or segmented styloid processes and associated them with cervicofacial symptoms in certain cases. Subsequently, Monsour and Young [2] expanded these observations through the systematic analysis of panoramic radiographs, highlighting the marked anatomical variability of the styloid complex in the general population.

One of the most widely accepted classifications was proposed by Langlais et al. [3], who incorporated both the morphology of the styloid process and its degree of mineralisation. This classification continues to be used in recent studies, reflecting its clinical usefulness and reproducibility [4,5].

From a diagnostic perspective, the evaluation of the styloid process can be performed using different imaging techniques. Several studies have demonstrated that panoramic radiography provides sufficient information to identify styloid process elongation (SPE) and to assess its morphological characteristics and mineralisation patterns [6,7]. Although cone-beam computed tomography (CBCT) offers more accurate three-dimensional visualisation and allows for more precise measurement of the actual length, its routine use is not always justified due to higher cost, increased resource utilisation, and greater exposure to ionising radiation [8,9,10]. For this reason, panoramic radiography remains the first-line imaging modality for the initial assessment of the styloid complex in daily clinical practice.

In orthodontic practice, panoramic radiography is part of the initial diagnostic protocol in adult patients seeking treatment. In addition to providing essential information on dental, periodontal, and osseous status, this imaging modality allows for the visualisation of anatomical structures adjacent to the dentoalveolar complex, including the styloid complex.

In this context, SPE is frequently identified as an incidental radiographic finding in patients who, in many cases, do not present clinical symptoms at the time of orthodontic evaluation.

The most widely accepted criterion for defining elongation is a length equal to or greater than 30 mm [11,12]. However, the presence of an SPE does not necessarily imply a clinically relevant condition. In certain cases, this elongation has been associated with Eagle syndrome, first described by Eagle in 1937, which may present in different clinical forms, including the classic type associated with a history of tonsillectomy and stylocarotid syndrome without prior surgical history. The associated symptomatology is heterogeneous and includes persistent pharyngeal pain, foreign body sensation, otalgia, dysphagia, cervical pain, and discomfort in the retromolar region [13,14].

Recent studies indicate that only a minority of patients with SPE present clinically relevant symptoms, with an estimated 4% to 10% of cases potentially consistent with Eagle syndrome [15,16]. In most patients, elongation represents an incidental finding detected during routine dental radiographic examinations, highlighting the importance of its correct clinical and radiological interpretation.

In adult patients seeking orthodontic treatment, the detection of SPE is of particular clinical relevance, as the subsequent onset of orofacial or cervical pain may be erroneously attributed to occlusal alterations, temporomandibular disorders, or muscular conditions. Early identification of SPE on panoramic radiographs may alert clinicians to the presence of an anatomical variation that should be considered when evaluating patients presenting with unexplained orofacial or cervical symptoms.

Despite the routine use of panoramic radiography and lateral cephalometric analysis in adult orthodontic patients, limited evidence exists regarding the relationship between styloid process elongation and craniofacial skeletal characteristics, particularly in European populations.

Therefore, the aim of the present study was to determine the prevalence and radiographic characteristics of styloid process elongation in adult orthodontic patients and to investigate its potential relationship with skeletal class, facial pattern, and clinically documented orofacial and cervical symptoms.

## 2. Materials and Methods

### 2.1. Study Design

A retrospective cross-sectional observational study was conducted in an adult population seeking orthodontic treatment who underwent panoramic radiography as part of the initial diagnostic assessment at the Faculty of Dentistry of the Complutense University of Madrid (Madrid, Spain) and at two private clinics.

**Figure 1:**
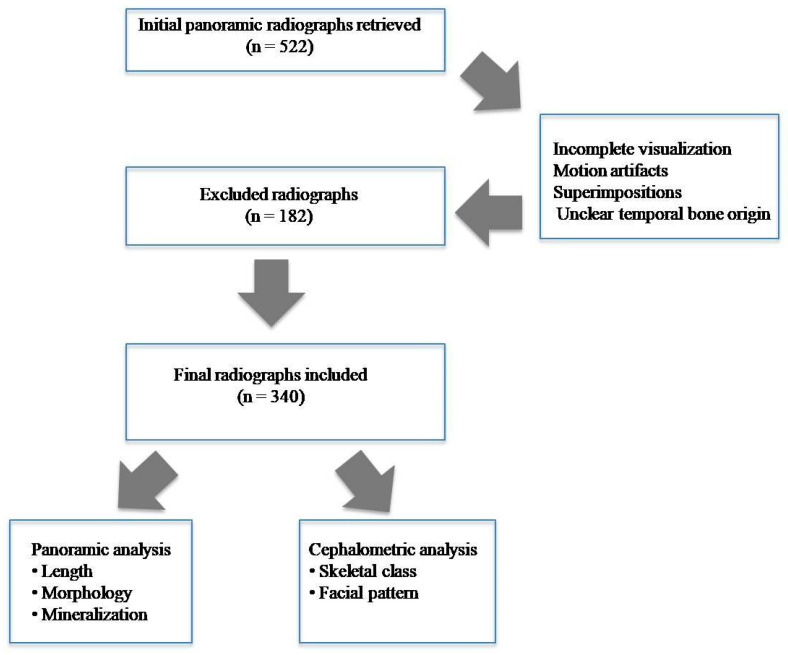
A flowchart illustrating the selection process of panoramic radiographs included in the study according to the STROBE recommendations.

A total of 522 panoramic radiographs were analysed, consecutively collected from adult patients seeking orthodontic treatment during the study period (October 2024 to July 2025), according to the availability of complete diagnostic records. Of these, 182 were excluded due to incomplete visualisation of the styloid process or its point of origin at the temporal bone. After applying these criteria, a final sample of 340 radiographs were included for analysis (Figure 1).

The study was reported in accordance with the Strengthening the Reporting of Observational Studies in Epidemiology (STROBE) guidelines [17].

The study protocol was approved by the Ethics Committee of Hospital Clínico San Carlos, Madrid, Spain (approval code: C.I. 21/497-E), on 20 July 2021 and was conducted in accordance with the principles of the Declaration of Helsinki. Written informed consent was obtained from all patients whose radiographic records were used for research purposes.

### 2.2. Radiographic Acquisition Protocol

All panoramic radiographs and lateral cephalograms were obtained using a Planmeca ProMax^®^ 3D Mid unit (Planmeca Oy, Helsinki, Finland), following the standardised positioning and exposure protocols recommended by the manufacturer and routinely applied in clinical practice at the participating centres.

All images were acquired by trained technical personnel and stored in digital format for subsequent analysis. All radiographs were obtained using the same imaging unit and standardised patient positioning protocols in order to minimise variations related to magnification and geometric distortion.

### 2.3. Radiograph Selection

Panoramic radiographs and lateral cephalograms were independently evaluated by two observers with experience in oral and maxillofacial radiology (N.M.-R. and M.A.-V.). The following inclusion criteria were established:

- Proper patient positioning and centring;
- Absence of motion artefacts or significant superimpositions;
- Complete visualisation of the styloid process along its entire course;
- Clear identification of the point of origin of the styloid process at the temporal bone.

Radiographs that did not meet these criteria were excluded from the study. Discrepancies between observers were resolved through discussion and consensus.

### 2.4. Observer Calibration

Prior to the final analysis, both observers participated in a pilot training and calibration session using 30 panoramic radiographs. The aim was to standardise the criteria for length measurement, as well as the morphological classification and mineralisation pattern assessment of the styloid process, in accordance with the classification proposed by Langlais et al. [3].

### 2.5. Measurement Protocol (Figure 2 and Figure 3)

Measurements were performed in millimetres using the measurement tools integrated within a digital radiographic viewing system (Weasis v3.8 DICOM Viewer) under standardised digital viewing conditions.

The length of the styloid process was measured using predefined anatomical landmarks. The proximal landmark was defined as the point of origin of the styloid process at the temporal bone, as visualised on panoramic radiography, whereas the distal landmark corresponded to the most inferior radiopaque tip identifiable along its anatomical axis.

In cases where the styloid process exhibited a curved morphology, a multipoint measurement tool was used to accurately follow its anatomical contour. For segmented styloid processes, the total length was measured as a continuous distance from the proximal origin to the distal tip, following the general anatomical axis.

All measurements were performed using standardised acquisition protocols and calibrated digital software tools to minimise potential variations related to magnification and geometric distortion inherent to panoramic radiography.

A length equal to or greater than 30 mm was adopted as the criterion for defining styloid process elongation, in accordance with previous research.

In cases of uncertainty regarding landmark identification or morphological classification, both observers jointly reviewed the radiographs and reached a consensus through discussion.

**Figure 2:**
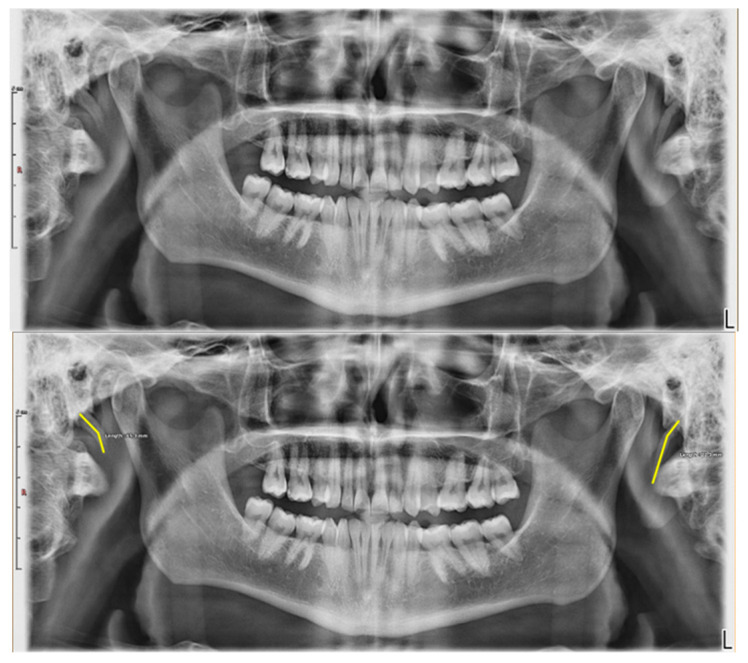
Panoramic radiographs showing styloid processes with normal length.

**Figure 3:**
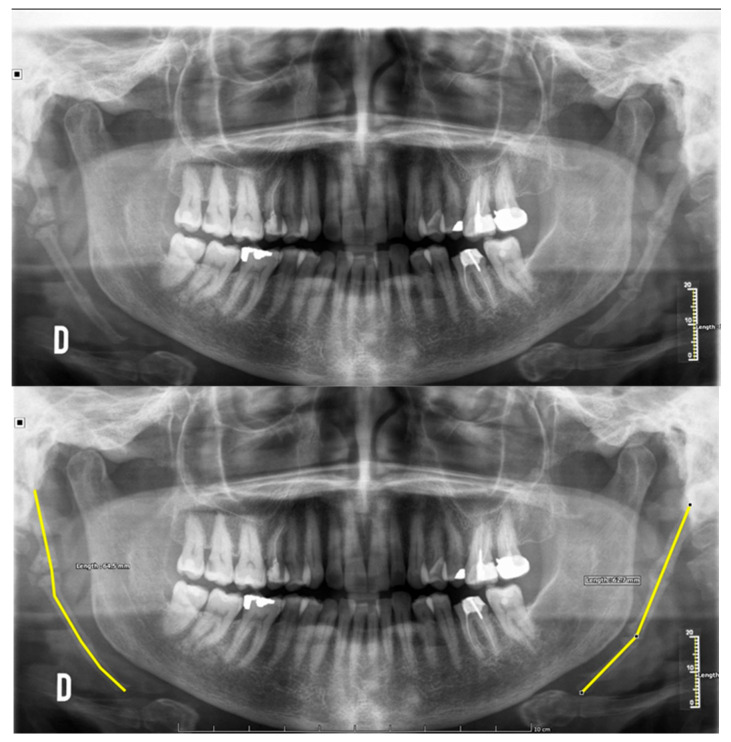
Panoramic radiographs showing elongated styloid processes.

### 2.6. Morphological Classification (Figure 4)

The morphology of the styloid process was classified according to the system proposed by Langlais et al. [3], with the addition of a fourth subtype to represent styloid processes of normal length and morphology. Four categories were established:

- Type I: elongated styloid process;
- Type II: pseudo-articulated styloid process;
- Type III: segmented styloid process with two or more segments;
- Type IV: styloid process of normal length and morphology.

**Figure 4:**
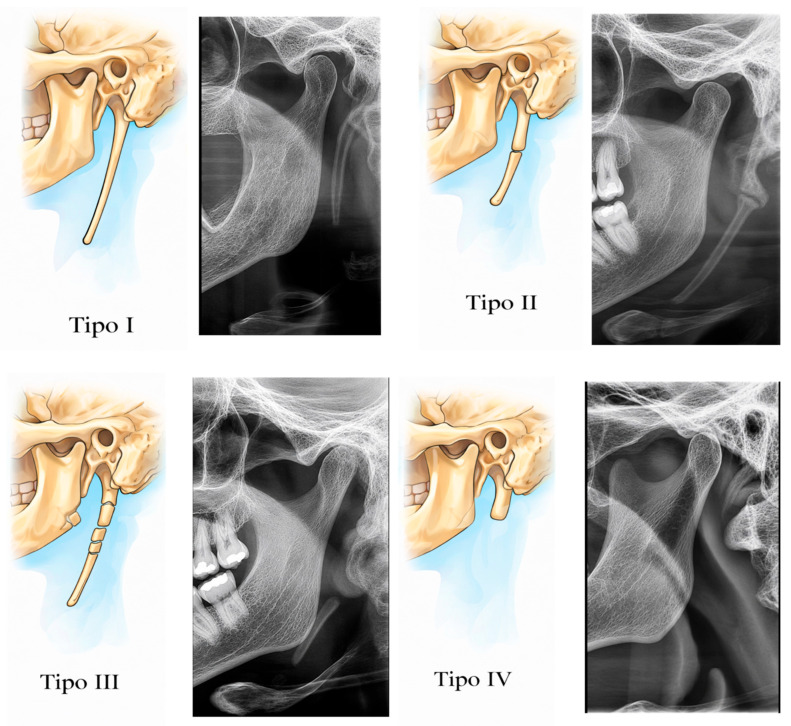
Morphological classification of the styloid process according to the modified Langlais classification.

### 2.7. Mineralisation Pattern Assessment (Figure 5)

The degree of mineralisation of the styloid process was assessed according to the system described by Langlais et al. [3], and classified into four types:

- Type A: mineralised outline with a radiolucent centre;
- Type B: partial mineralisation with discontinuous radiolucent areas;
- Type C: nodular pattern with variations in central mineralisation;
- Type D: complete mineralisation.

**Figure 5:**
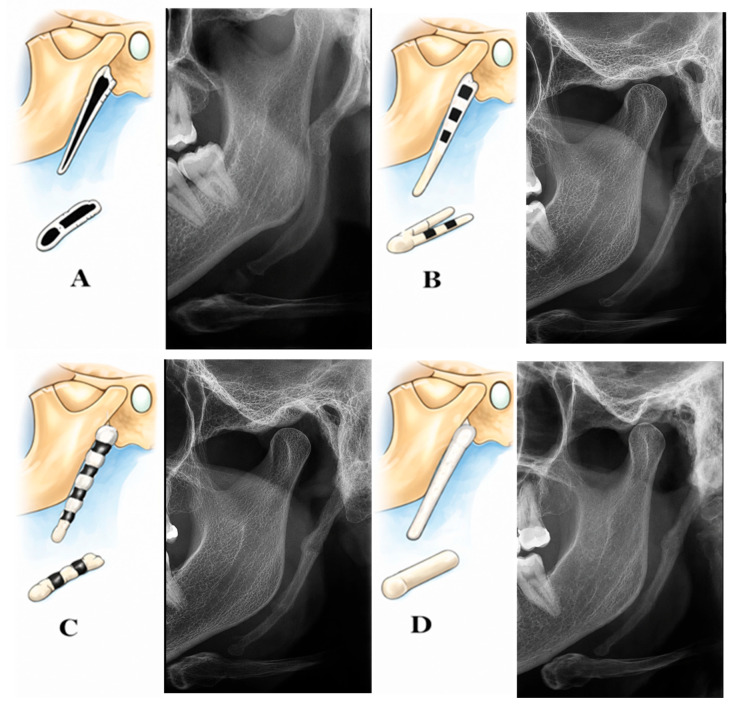
Mineralisation patterns of the styloid process according to the Langlais classification.

### 2.8. Symmetry Assessment

Bilateral symmetry was defined as the presence of similar morphology and comparable lengths on both sides, allowing for a maximum difference of ±5 mm between the right and left styloid processes [18,19]. This criterion was used for descriptive and comparative purposes.

### 2.9. Assessment of Skeletal Class and Facial Pattern

Skeletal class and facial pattern were assessed using lateral cephalometric radiographs obtained as part of the initial orthodontic diagnostic records.

Skeletal classification was determined using Steiner’s ANB angle, defined as a cephalometric parameter that evaluates the anteroposterior relationship between the maxilla (Point A) and the mandible (Point B) with respect to the anterior cranial base (Nasion). Patients were classified as skeletal Class I (ANB between 0° and 4°), skeletal Class II (ANB > 4°), and orskeletal Class III (ANB < 0°), according to the criteria described by Steiner [20].

Additionally, Ricketts’ facial convexity was recorded as a complementary parameter for the evaluation of the sagittal maxillomandibular relationship. Positive values indicate a convex skeletal Class II profile, values of 0 mm correspond to the skeletal Class I pattern described by Ricketts, and negative values indicate a tendency toward a concave skeletal Class III profile [21].

Vertical facial pattern was determined according to mandibular inclination relative to the cranial base using the SN-GoGn angle. Patients were classified as brachyfacial (SN-GoGn < 28°), mesofacial (SN-GoGn between 28° and 36°), or dolichofacial (SN-GoGn > 36°), following the cephalometric criteria established in the literature [22,23].

All measurements were performed by previously calibrated observers, following standardised analysis criteria.

### 2.10. Intra- and Interobserver Agreement Analysis

To assess the reproducibility of the measurements and classifications, a total of fifty panoramic radiographs and lateral cephalometric radiographs were randomly selected from the overall sample. Interobserver agreement was evaluated by comparing the classifications independently performed by both observers (N.M.-R. and M.A.-V.).

Agreement for categorical variables (elongation, morphology, mineralisation pattern, symmetry, skeletal class, and facial pattern) was assessed using Cohen’s kappa coefficient and interpreted according to the criteria proposed by Landis and Koch.

### 2.11. Clinical Data Collection

Following the final radiographic analysis, two additional observers (C.M.-R. and J.A.-F.) reviewed the patients’ clinical records to identify cases in which any signs or symptoms within the orofacial and cervical regions had been documented.

### 2.12. Data Analysis and Statistics

Statistical analysis was performed using SPSS 31 software (Statistical Package for the Social Sciences). Categorical variables were expressed as absolute frequencies and percentages and were analysed using the chi-square test. Continuous variables were assessed using the Mann–Whitney U test due to their non-normal distribution. Symmetry between the values obtained on the right and left sides was evaluated using the Wilcoxon signed-rank test for related samples. Finally, agreement between morphological and mineralization patterns on both sides was assessed using Cohen’s kappa test and McNemar’s test. Statistical significance was set at *p* < 0.05.

ChatGPT Pro 5.5. (OpenAI) was used as an assistive tool for the preparation and design of the figures to improve their visual presentation and organisation. The use of this tool was limited exclusively to figure design and did not contribute to data collection, statistical analysis, interpretation of the results, or the study conclusions.

## 3. Results

### 3.1. Sample Characteristics

The sample consisted of 130 males (38.24%) with a mean age of 50.74 ± 6.60 years and 210 females (61.76%) with a mean age of 52.06 ± 6.59 years. The age difference between males and females showed a small effect size (Cohen’s d = 0.20), indicating minimal differences between the two groups.

### 3.2. Interobserver Agreement

The assessment of reproducibility demonstrated good interobserver agreement for all variables analysed.

Agreement for the morphological classification of the styloid process was κ = 0.76, while a slightly higher value was observed for the identification of mineralisation patterns (κ = 0.82). The measurement of styloid process length showed an even higher agreement, with a kappa value of κ = 0.88.

### 3.3. Length of the Styloid Processes (Table 1)

Measurements of styloid process length showed that 178 patients (52.35%) had values < 30 mm, whereas elongation (>30 mm) was observed in 162 patients (47.65%) (Figure 3 and Figure 4).

**Table 1:** Descriptive statistics of age and styloid process length according to the presence or absence of elongation.

			*N*	*Mean*	*Standard Deviation*	*Minimum*	*Maximum*
*Age*	Elongation	No	178	51.58	5.96	40.00	68.00
		Yes	162	51.52	7.29	40.00	70.00
		Total	340	51.55	6.62	40.00	70.00
*Length Right*	Elongation	No	178	23.93	3.26	19.06	29.87
		Yes	162	51.16	12.08	30.05	69.54
		Total	340	36.90	16.14	19.06	69.54
*Length Left*	Elongation	No	178	24.88	4.04	19.27	68.94
		Yes	162	39.35	15.44	19.00	69.54
		Total	340	35.97	15.05	19.00	69.54

In patients without elongation, the mean length was 23.93 ± 3.26 mm on the right side and 24.88 ± 4.04 mm on the left side, with no statistically significant differences between sides (*p* = 0.621), suggesting a symmetrical distribution.

In contrast, in patients with elongation, the mean length on the right side was 51.16 ± 12.08 mm, compared to 39.35 ± 15.44 mm on the left side, indicating asymmetry between both styloid processes (*p* = 0.001).

**Figure 6:**
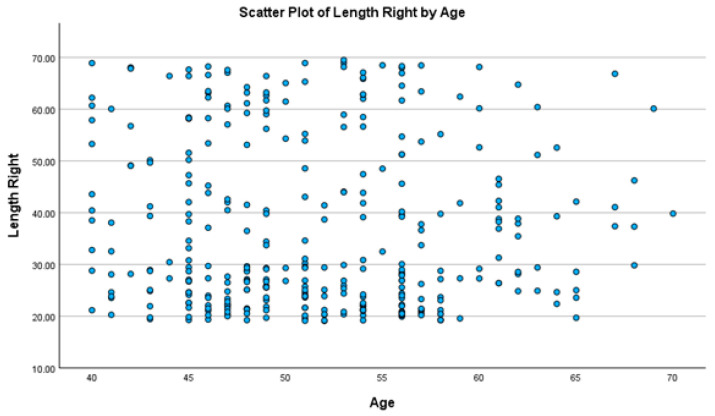
Correlation analysis between age and right SPE.

**Figure 7:**
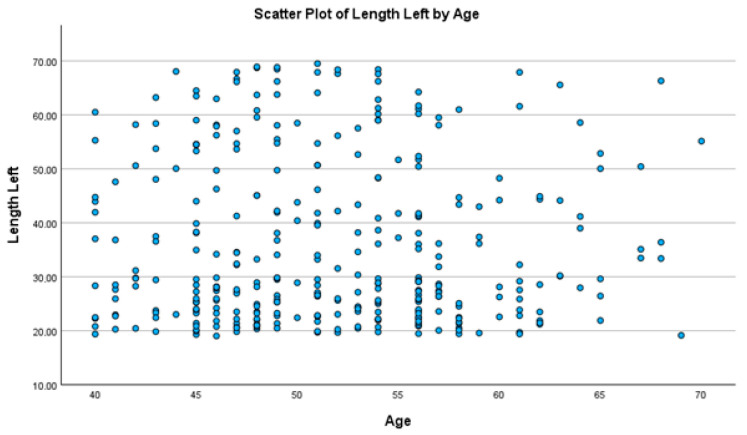
Correlation analysis between age and left SPE.

The correlation analysis between age and SPE was not statistically significant, with *p*-values of 0.235 for the right side and 0.978 for the left side (Figure 6 and Figure 7).

**Figure 8:**
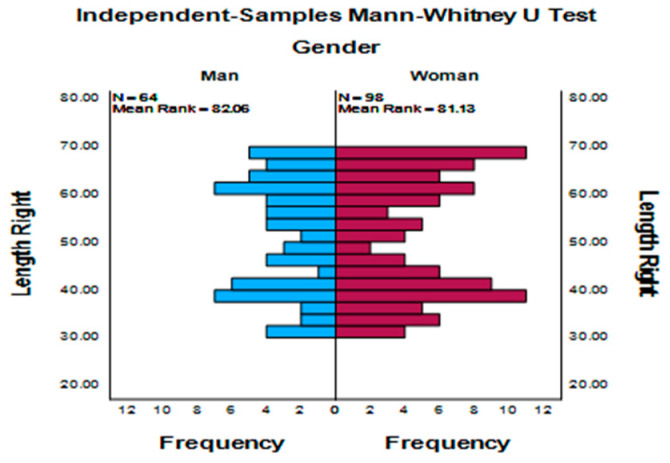
Distribution of right SPE according to sex.

**Figure 9:**
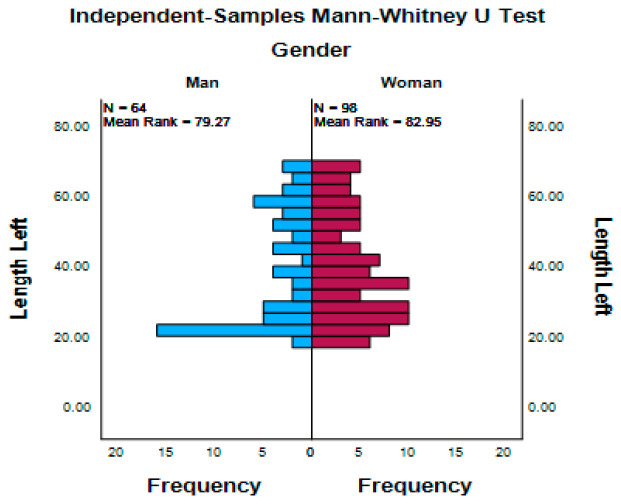
Distribution of left SPE according to sex.

Similarly, no statistically significant association was found between sex and SPE, either on the right side (*p* = 0.902) or the left side (*p* = 0.625) (Figure 8 and Figure 9).

### 3.4. Morphological and Mineralisation Patterns

Analysis of the different morphological patterns of the styloid processes (Table 2) showed that the normal morphology pattern (Type IV) was the most frequent, followed in decreasing order by the pseudo-articulated type (Type II), the elongated type (Type I), and the segmented type (Type III).

The analysis of agreement between the right and left sides regarding the different morphological patterns showed an agreement rate of 55% (Kappa = 0.272; McNemar test: *p* = 0.048).

Comparison of morphological patterns by sex revealed no significant differences on either side (Table 3 and Table 4).

Analysis of the different mineralisation patterns (Table 5) showed that the complete type (Type D) was the most frequent, followed by the discontinuous type (Type B), the type without internal calcifications (Type A), and the nodular type (Type C).

Similar to the morphological patterns, mineralisation patterns did not show statistically significant differences between the right and left sides.

Agreement analysis between the right and left sides for the different mineralization patterns demonstrated a 93.28% agreement (Kappa = 0.899; McNemar test: *p* < 0.001).

Unlike the morphologies, significant differences were found when comparing morphological patterns according to sex (Table 6 and Table 7).

### 3.5. Skeletal Class and Facial Pattern

Analysis of the distribution of SPE according to skeletal class (Table 8) showed that the highest proportion of cases corresponded to Class II patients, followed by Class I and Class III.

In terms of relative frequency, elongation was slightly more prevalent in Class I patients (52.1%), whereas lower percentages were observed in Class II and Class III patients (45.8% and 44.1%, respectively). However, these differences did not show a clear trend suggesting a significant association between SPE and skeletal class (*p* = 0.479).

Regarding facial pattern (Table 9), the sample distribution showed a predominance of brachyfacial patients, followed by mesofacial and dolichofacial individuals. The highest proportion of elongation was observed in mesofacial patients (51.3%), followed by brachyfacial (47.7%) and dolichofacial patients (43.9%).

Similar to the skeletal class analysis, no consistent association was found between facial pattern and the presence of SPE, with a relatively homogeneous distribution across the different facial types (*p* = 0.531).

### 3.6. Clinical Findings

A review of the clinical records revealed that only 15 patients reported having experienced intermittent signs or symptoms, including joint noises (4.41%), tinnitus (2.94%), ear pain (2.05%), and headaches (3.23%).

## 4. Discussion

The present study analyses SPE as an incidental radiographic finding in an adult population seeking orthodontic treatment and evaluated by means of panoramic radiography as part of the initial diagnostic assessment. Our results show that more than half of the patients presented at least one SPE, confirming that this anatomical variation is not uncommon in routine dental and orthodontic practice, even in the absence of clinically relevant symptoms.

Reported prevalence rates in the literature vary widely, ranging from 12.64% to 72.75%, depending on the population studied, the imaging modality used, and the criteria applied to define elongation [5,18,24,25,26].

Several systematic reviews have highlighted that this methodological heterogeneity represents one of the main factors responsible for the variability in published results, which may hinder direct comparison between studies [27,28,29].

In the present study, the prevalence of elongation was 47.65% in adult patients evaluated for orthodontic treatment, a value higher than that reported in many general populations, which may be related to the age of the sample and the specific clinical setting analysed [30,31].

This finding is particularly relevant in orthodontics, where an increasing number of adult patients are systematically evaluated using panoramic radiography.

Another noteworthy finding was the high proportion of bilateral elongations, with frequent symmetry in length and/or morphology between both sides. This pattern has been previously described in population-based radiographic studies and highlights that SPE does not typically present as a uniform or symmetrical alteration [31,32,33]. Nevertheless, from a clinical perspective, the possibility of anatomical variability supports the need for individualised assessment of both sides of the styloid complex during the interpretation of panoramic radiographs.

Regarding morphological patterns, the results showed a predominance of styloid processes with normal length and morphology (Type IV) and pseudo-articulated processes (Type II), whereas continuous elongations (Type I) and segmented patterns (Type III) were less frequent. These findings are consistent with recent panoramic radiography-based studies that describe a wide morphological variability and emphasise that SPE does not follow a single pattern in adult populations [34,35,36].

With respect to the degree of mineralisation, the most frequent pattern observed in our study was complete mineralisation (Type D), followed by partially mineralised patterns. This finding is consistent with the hypothesis that ossification of the stylohyoid complex progresses with age and exhibits marked individual variability [37,38].

Regarding the potential association between SPE and cephalometric variables, the results of the present study did not demonstrate a statistically significant relationship with either skeletal class or facial pattern. These findings partially contrast with some previous studies that have suggested a greater length or prevalence of elongation in Class II patients or in hyperdivergent facial patterns. However, the available evidence remains limited and heterogeneous, with important differences in study design, sample size, age range, and, particularly, in measurement methodologies and diagnostic criteria [5,39,40].

In this context, the lack of association observed in our sample may be explained by the multifactorial nature of styloid elongation, in which factors such as age, individual anatomical variability, and ossification processes of the stylohyoid complex may play a more relevant role than craniofacial skeletal characteristics. Furthermore, the inclusion of an adult orthodontic population with a relatively balanced distribution of facial biotypes may have contributed to the reduced influence of cephalometric variables on this finding.

Therefore, the results of this study suggest that SPE should be primarily considered an anatomical variation independent of skeletal morphology. Nevertheless, further prospective studies, particularly those incorporating three-dimensional imaging techniques, are needed to provide more robust evidence regarding this potential relationship.

Another aspect of this study was to explore, particularly in cases of SPE, the presence of clinical manifestations suggestive of Eagle syndrome.

Several studies have indicated that the presence of calcification or advanced mineralisation should not be automatically interpreted as a pathological finding, as it may be observed in asymptomatic individuals. Only a minority of patients with styloid process elongation develop clinically relevant symptoms, with approximately 4% to 10% of elongated cases presenting features consistent with Eagle syndrome [13,18,29,30,32,41].

In our study, only 15 patients reported having experienced symptoms at some point, which were more consistent with temporomandibular disorders than with Eagle syndrome.

It should be emphasised that the present study was not designed to establish a direct causal relationship between styloid process elongation and specific orofacial pain conditions, nor to evaluate treatment outcomes. Rather, its primary objective was to determine the prevalence and radiographic characteristics of this anatomical variation in an adult orthodontic population. Consequently, the clinical implications of styloid process elongation should be interpreted with caution, and future prospective studies are needed to clarify its potential role in the development of orofacial and cervical symptoms.

Nevertheless, this aspect is particularly relevant in orthodontic practice, where patients may present with orofacial pain, headaches, cervical pain, or otalgia during treatment. These symptoms may be erroneously attributed to temporomandibular dysfunction, muscular disorders, or occlusal alterations [42,43].

Therefore, the early identification of SPE on the initial panoramic radiograph may be crucial for orthodontists when establishing a differential diagnosis between these conditions, thereby optimising clinical decision-making and avoiding unnecessary investigations.

From a clinical perspective, the findings of the present study reinforce the importance of carefully evaluating the styloid complex during routine orthodontic radiographic assessment. Styloid process elongation should not be interpreted exclusively as an anatomical curiosity, but rather as a potentially relevant incidental finding that may contribute to the differential diagnosis of orofacial pain, cervical discomfort, otalgia, or temporomandibular-related symptoms.

In this context, panoramic radiography provides orthodontists with a valuable opportunity for the early recognition of this anatomical variation during routine diagnostic assessments, potentially reducing the risk of misdiagnosis and unnecessary therapeutic interventions.

Furthermore, the present study may serve as an initial reference framework for future multicentre and multiethnic investigations aimed at evaluating the clinical significance of styloid process elongation across different populations.

Finally, several limitations of the present study should be acknowledged, including its retrospective design and the exclusive use of panoramic radiography, which may affect the absolute accuracy of length measurements despite the high level of agreement obtained. In addition, panoramic radiography does not allow for an accurate three-dimensional assessment of the angulation and spatial orientation of the styloid process, factors that may influence the development of orofacial symptoms.

Moreover, clinical information obtained from patient records may underestimate the presence of mild or intermittent symptoms. Therefore, future prospective studies incorporating CBCT imaging and standardised clinical evaluation protocols could provide a more comprehensive assessment of the anatomical and clinical significance of styloid process elongation.

## 5. Conclusions

Styloid process elongation was a frequent incidental radiographic finding in the adult orthodontic patients included in the present study, with a prevalence of 47.65%. Considerable variability was observed in both morphology and mineralization patterns, with normal morphology (Type IV) and complete mineralization (Type D) being the most prevalent findings.

No statistically significant association was identified between styloid process elongation and skeletal class or facial pattern, suggesting that this anatomical variation may be independent of craniofacial skeletal morphology.

Furthermore, only a small proportion of patients reported symptoms according to the available clinical records, indicating that styloid process elongation does not necessarily represent a clinically relevant condition or imply the presence of Eagle syndrome.

The routine assessment of the styloid complex on panoramic radiographs may contribute to the differential diagnosis of orofacial and cervical pain during orthodontic evaluation and may help reduce potential diagnostic errors.

## Figures and Tables

**Table 2:** Comparison of the different morphological types of the right and left styloid processes.

			Type I	Type II	Type III	Type IV	Total	Chi-Square Value	*p* Value	Contingency Coefficient
SIDE	Right	Count	41	115	6	178	340	7.248	0.064	0.103
		% within SIDE	12.1%	33.8%	1.8%	52.4%	100.0%			
	Left	Count	60	90	9	181	340			
		% within SIDE	17.6%	26.5%	2.6%	53.2%	100.0%			
Total		Count	101	205	15	359	680			
		% within SIDE	14.9%	30.1%	2.2%	52.8%	100.0%			

**Table 3:** Comparison of different right-side morphological types by sex.

			Type I	Type II	Type III	Type IV	Total	Chi-Square Value	*p* Value	Contingency Coefficient
Sex	Man	Count	20	40	4	66	130	4.666	0.198	0.116
		% within Sex	15.4%	30.8%	3.1%	50.8%	100%			
	Woman	Count	21	75	2	112	210			
		% within Sex	10.0%	35.7%	1.0%	53.3%	100%			
Total		Count	41	115	6	178	340			
		% within Sex	12.1%	33.8%	1.8%	52.4%	100%			

**Table 4:** Comparison of different left-side morphological types by sex.

			Type I	Type II	Type III	Type IV	Total	Chi-Square Value	*p* Value	Contingency Coefficient
Sex	Man	Count	26	32	4	68	130	1.115	0.773	0.057
		% within Sex	20.0%	24.6%	3.1%	52.3%	100%			
	Woman	Count	34	58	5	113	210			
		% within Sex	16.2%	27.6%	2.4%	53.8%	100%			
Total		Count	60	90	9	181	340			
		% within Sex	17.6%	265%	2.6%	53.2%	100%			

**Table 5:** Comparison of mineralisation patterns between the right and left styloid processes.

			Type C	Type A	Type B	Type D	Total	Chi-Square Value	*p* Value	Contingency Coefficient
SIDE	Right	Count	12	80	113	135	340	3.189	0.363	0.068
		% within SIDE	3.5%	23.5%	33.2%	39.7%	100%			
	Left	Count	11	70	101	158	340			
		% within SIDE	3.2%	20.6%	29.7%	46.5%	100%			
Total		Count	23	150	214	293	680			
		% within SIDE	3.4%	22.1%	31.5%	43.1%	100.0%			

**Table 6:** Comparison of different right-side mineralisation types by sex.

			Type C	Type A	Type B	Type D	Total	Chi-Square Value	*p* Value	Contingency Coefficient
Sex	Man	Count	4	32	30	64	130	11.572	0.009	0.181
		% within Sex	3.1%	24.6%	23.1%	49.2%	100%			
	Woman	Count	8	48	83	71	210			
		% within Sex	3.8%	22.9%	39.5%	33.8%	100%			
Total		Count	12	80	113	135	340			
		% within Sex	3.5%	23.5%	33.2%	39.7%	100%			

**Table 7:** Comparison of different left-side mineralisation types by sex.

			Type C	Type A	Type B	Type D	Total	Chi-Square Value	*p* Value	Contingency Coefficient
Sex	Man	Count	4	28	26	72	130	10.382	0.016	0.172
		% within Sex	3.1%	21.5%	20.0%	55.4%	100%			
	Woman	Count	7	42	75	86	210			
		% within Sex	3.3%	20.0%	35.7%	41.0%	100%			
Total		Count	11	70	101	158	340			
		% within Sex	3.2%	20.6%	29.7%	46.5%	100%			

**Table 8:** Comparison of skeletal classes.

Squeletal Class	Number of Cases	No Elongation (%)	Yes Elongation (%)
Class 1	119	57 (47.9%)	62 (52.1%)
Class 2	153	83 (54.2)	70 (45.8%)
Class 3	68	38 (55.9%)	30 (44.1%)
Total	340	178	162

**Table 9:** Distribution of facial patterns and their association with SPE.

Facial Pattern	Number of Cases	No Elongation (%)	Yes Elongation (%)
Mesofacial	119	58 (48.7%)	61 (51.3%)
Brachyfacial	130	68 (52.3%)	62 (47.7%)
Dolichofacial	91	51 (56.1%)	40 (43.9%)
Total	340	177	163

## Data Availability

The data presented in this study are available from the corresponding author upon reasonable request.
